# Supplementary material for: Lack of an atypical PDR transporter generates an immunogenic Cryptococcus neoformans strain that drives a dysregulated and lethal immune response in murine lungs
Source: mBio. 2025 May 30;16(7):e01321-25. doi: 10.1128/mbio.01321-25 (PMC12239598; doi:10.1128/mbio.01321-25)
Supplement: Supplemental Figures — Figures S1 to S5. [file mbio.01321-25-s0001.pdf]

Supplemental Material for

Lack of an atypical PDR transporter generates an immunogenic *Cryptococcus neoformans* strain that drives a dysregulated and lethal immune response in murine lungs

Christopher J. Winski, Peter V. Stuckey, Armando M. Marrufo, Georgina Agyei, Robbi L. Ross, Tamanna Urmi, Sarah Chapman, and Felipe H. Santiago-Tirado

Figure S1

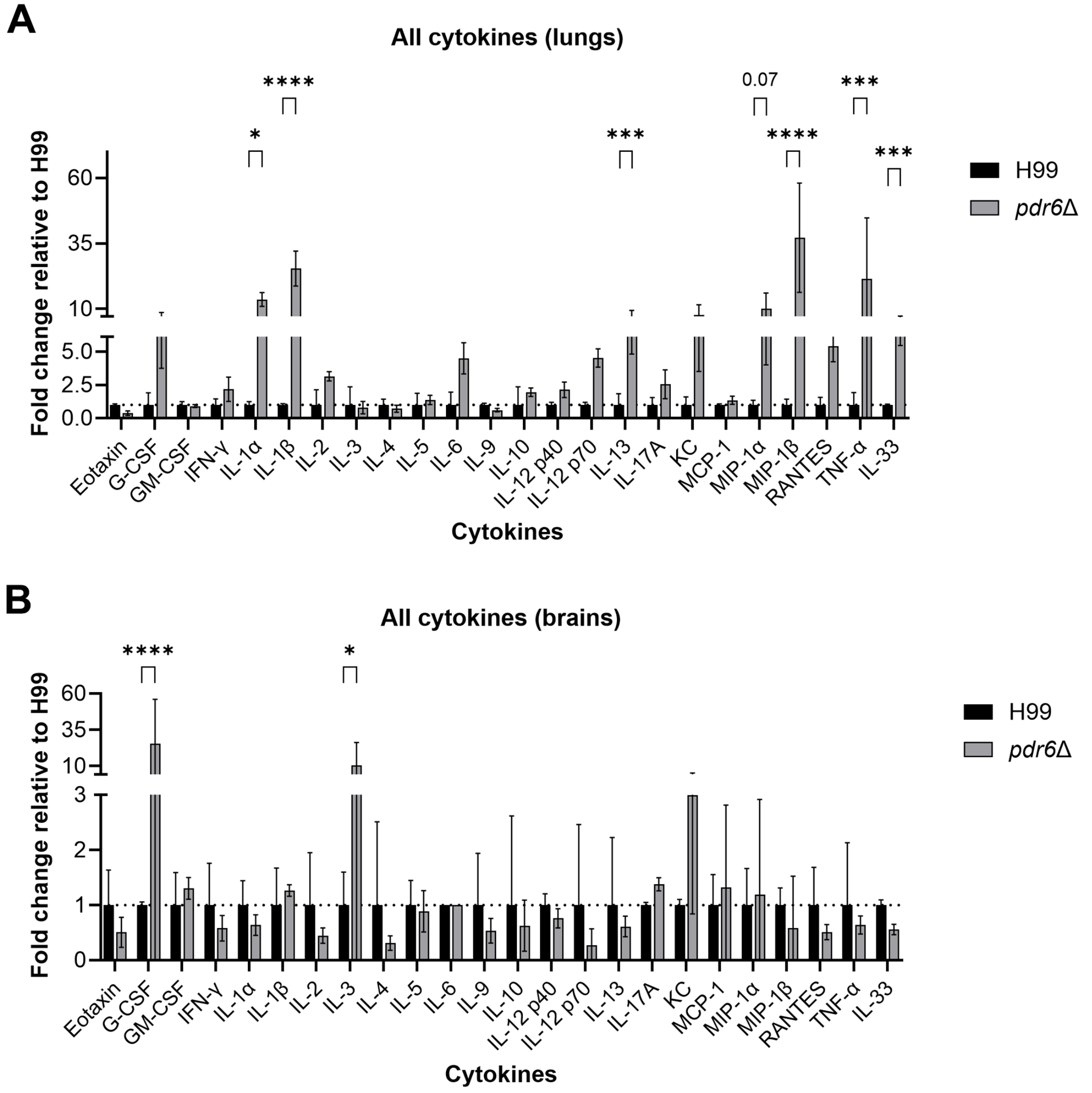

Fig. S1 Cytokine/chemokine levels in the lungs (A) and brains (B) of *pdr6*Δ-infected mice relative to the levels in H99-infected mice. Significance was determined by Ordinary 2-way ANOVA with multiple comparisons, *P<0.05; **P<0.01; ***P<0.001; ****P<0.0001.

Figure S2

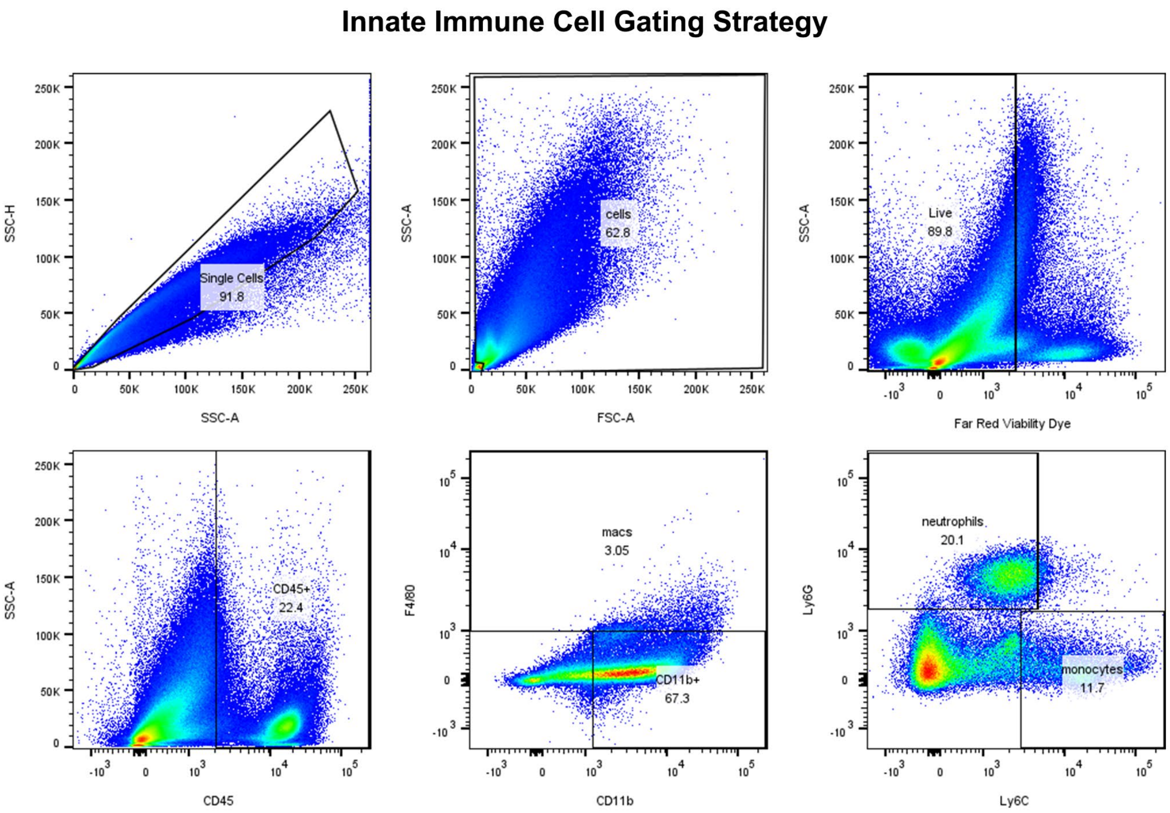
Innate Immune Cell Gating Strategy
Single Cells
91.8
SSC-H
SSC-A
cells
62.8
SSC-A
FSC-A
Live
89.8
SSC-A
Far Red Viability Dye
CD45+
22.4
SSC-A
CD45
macs
3.05
CD11b+
67.3
F4/80
CD11b
neutrophils
20.1
monocytes
11.7
Ly6G
Ly6C

Adaptive Immune Cell Gating Strategy

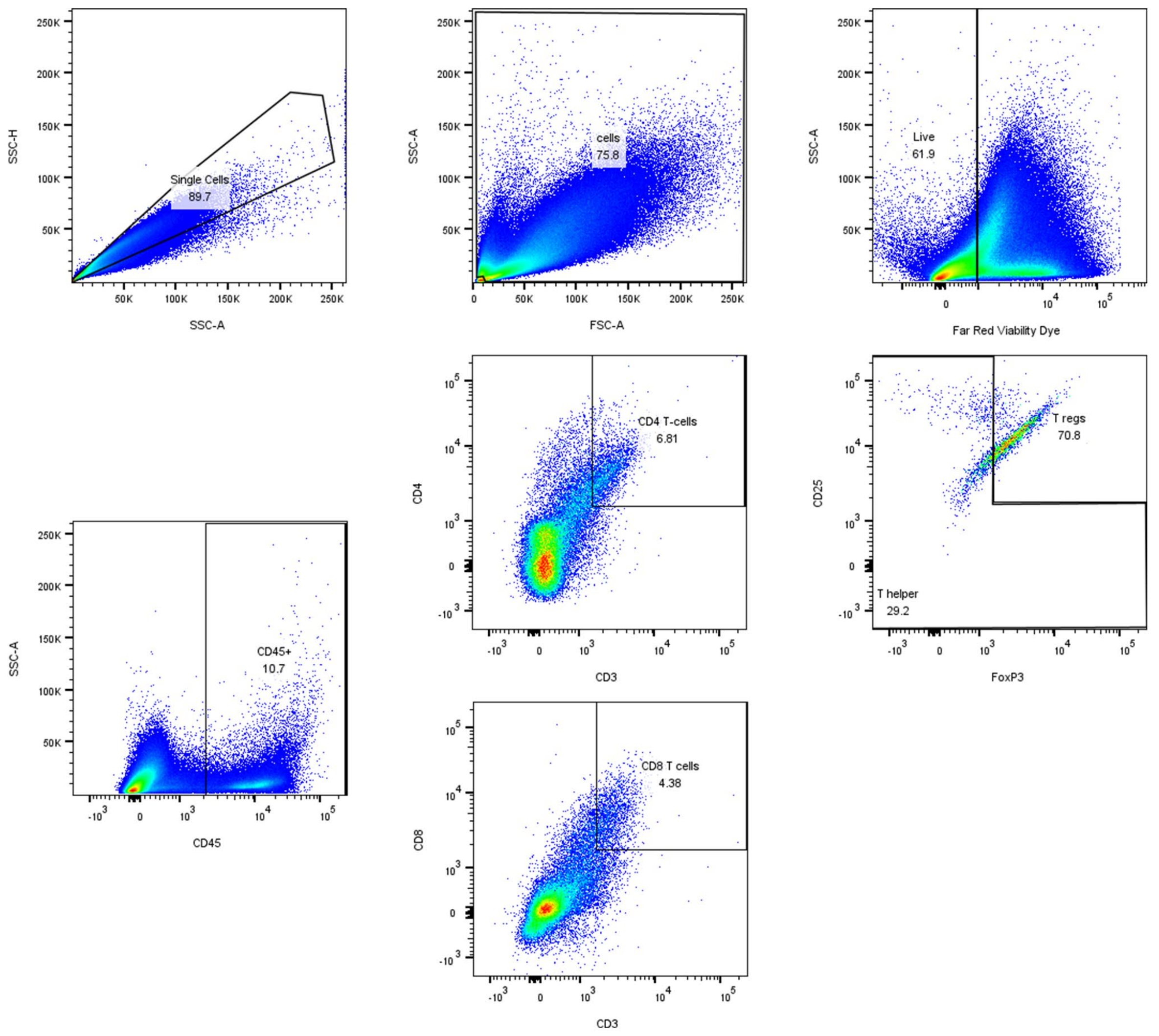

Fig. S2 Example of the cell gating strategy used to quantify the different immune cell populations. Monocytes (CD45+F4/80-CD11b+Ly6C+), neutrophils (CD45+F4/80-CD11b+Ly6G+), macrophages (CD45+F4/80+CD11b+), CD4^{+} T-helper (CD45+CD3+CD4+), CD4^{+} T-regs (CD45+CD3+CD4+CD25+FoxP3+), and CD8^{+} T-helper (CD45+CD3+CD8+) cells.

Figure S3

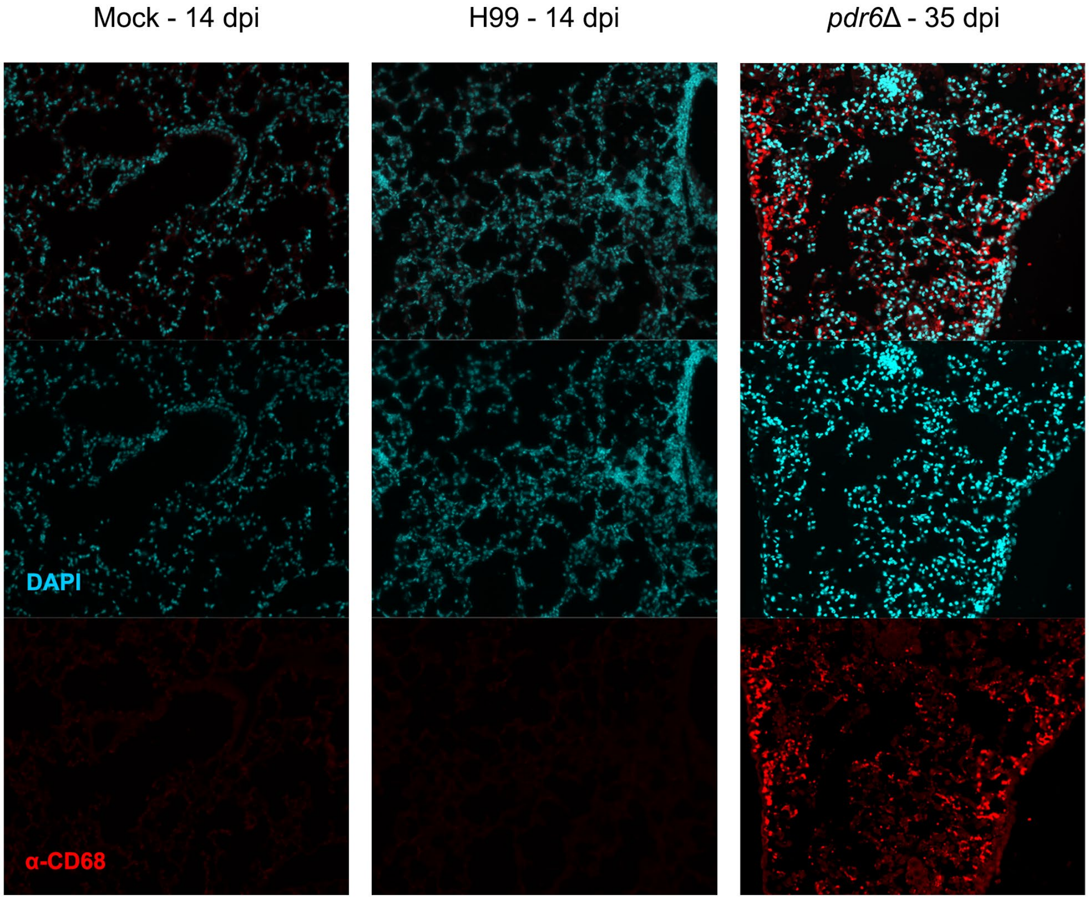

Fig. S3 Representative immunohistochemistry images of CD68+ cells in the lungs of naive and infected A/J mice. A/J mice were infected intranasally with *C. neoformans* H99 or *pdr6*Δ mutant, or mock infected with PBS, and euthanized for histological analysis at 14 (H99) or 35 (*pdr6*Δ mutant) dpi. CD68 is shown in red (anti-CD68 antibody) while nuclei is shown in cyan (stained with DAPI). The top row is a merge of the individual channels shown in the middle and bottom rows.

Figure S4

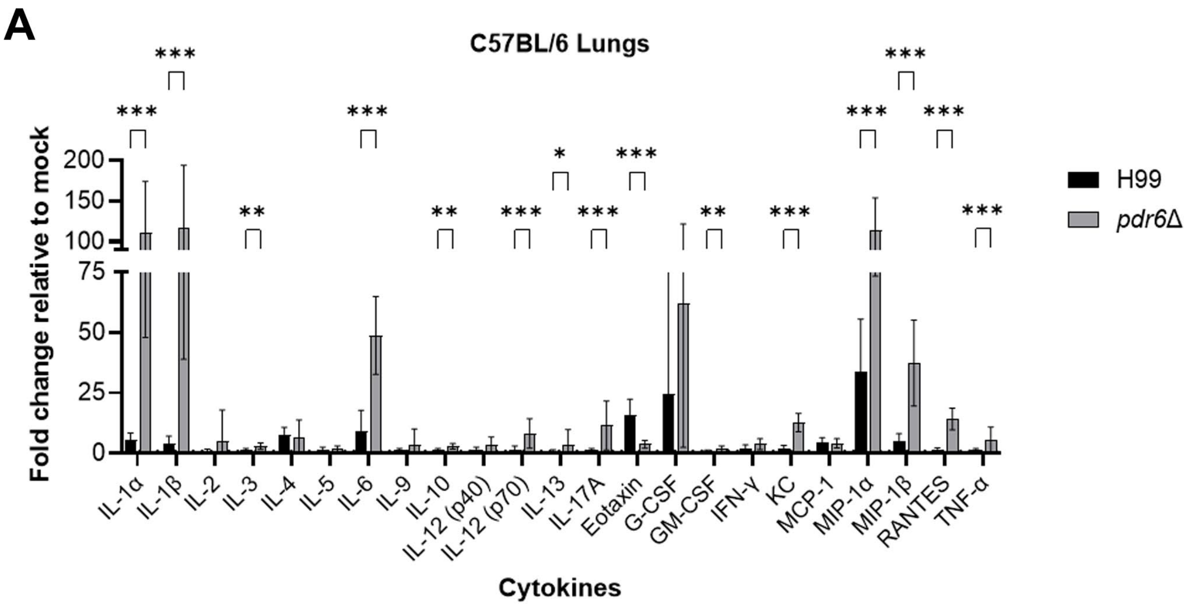
A
C57BL/6 Lungs
Fold change relative to mock
Cytokines
H99
pdr6Δ
IL-1α
IL-1β
IL-2
IL-3
IL-4
IL-5
IL-6
IL-9
IL-10
IL-12 (p40)
IL-12 (p70)
IL-13
IL-17A
Eotaxin
G-CSF
GM-CSF
IFN-γ
KC
MCP-1
MIP-1α
MIP-1β
RANTES
TNF-α

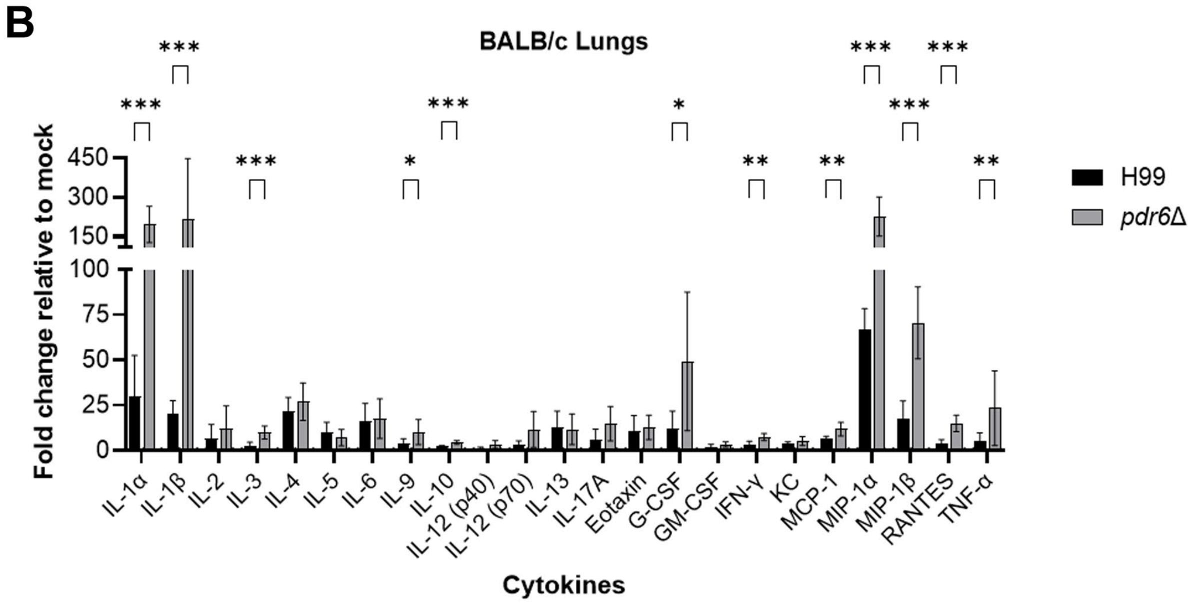
B
BALB/c Lungs
Fold change relative to mock
Cytokines
H99
pdr6Δ
IL-1α
IL-1β
IL-2
IL-3
IL-4
IL-5
IL-6
IL-9
IL-10
IL-12 (p40)
IL-12 (p70)
IL-13
IL-17A
Eotaxin
G-CSF
GM-CSF
IFN-γ
KC
MCP-1
MIP-1α
MIP-1β
RANTES
TNF-α

C

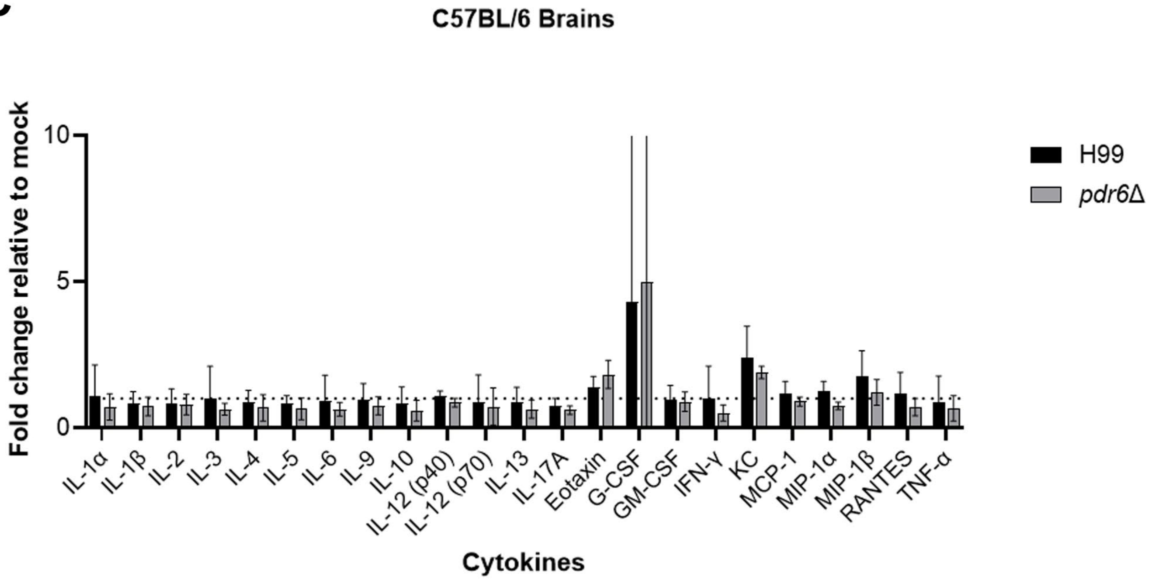

D

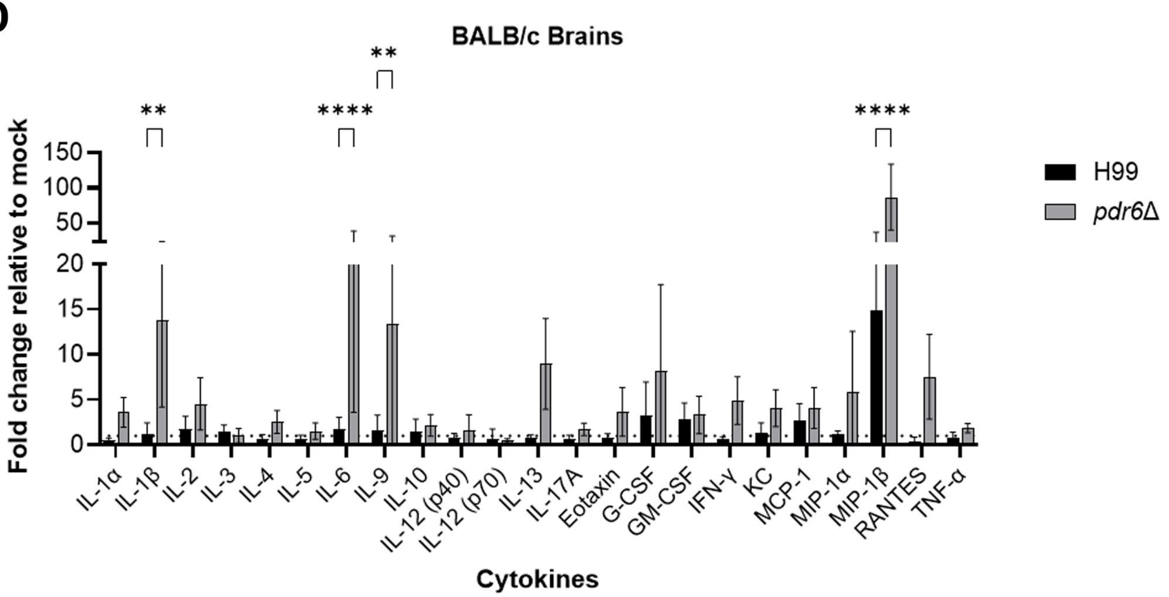

Fig. S4 Cytokine/chemokine levels in the lungs (A & B) and brains (C & D) of mice with skewed immune responses infected with *C. neoformans*. C57BL/6 mice **(A & C)** or BALB/c mice **(B & D)** were intranasally infected with 5 x 10^4 H99 or *pdr6*Δ cells and euthanized at endpoint for cytokine/chemokine analysis. The levels are shown relative to the levels in naïve (mock-infected)

mice. Significance was determined by Ordinary 2-way ANOVA with multiple comparisons, *P<0.05; **P<0.01; ***P<0.001; ****P<0.0001.

Figure S5

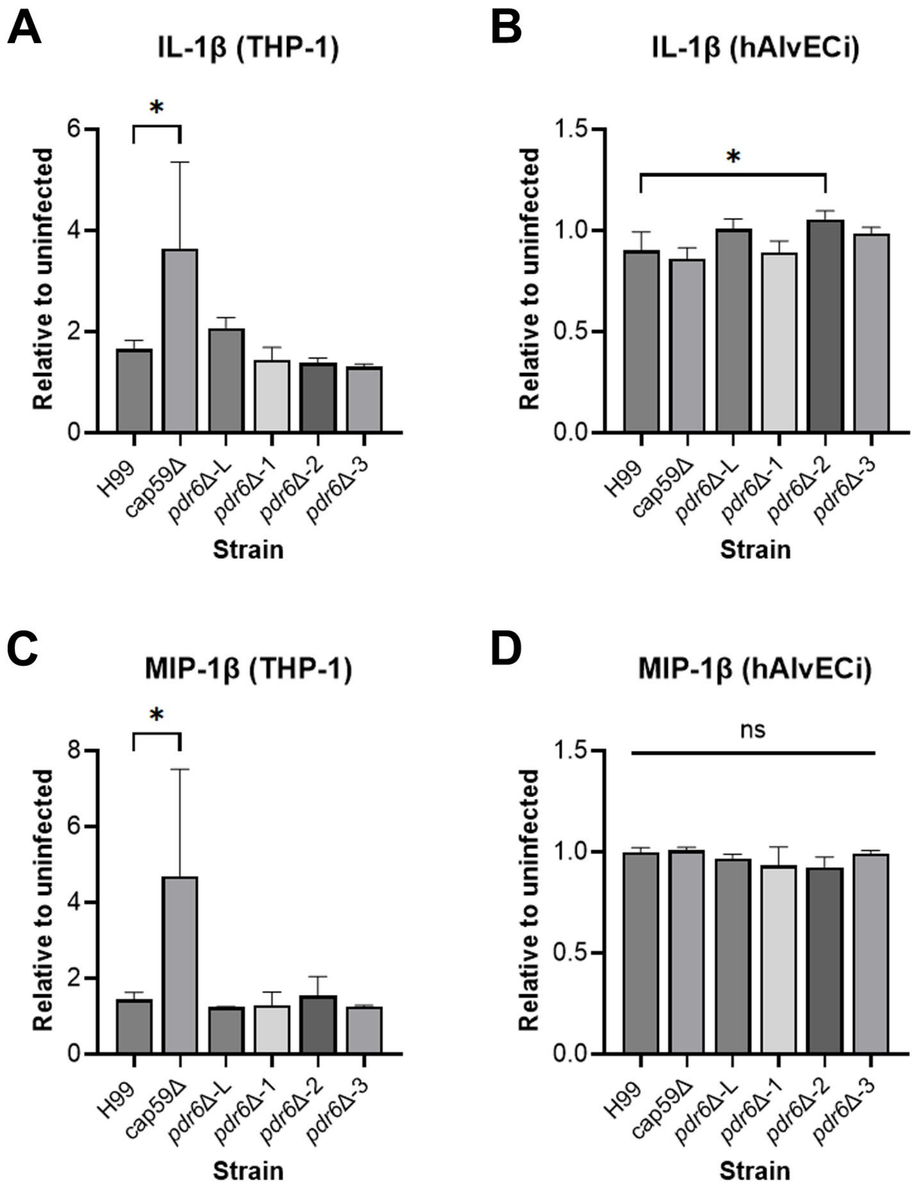

Fig. S5 Quantification of IL-1β and MIP-1β in the supernatant of THP-1 or hAlvECi cells exposed to *C. neoformans*. The indicated cells (THP-1 and hAlvECi are human cell lines representing alveolar macrophages and alveolar epithelial cells, respectively) were incubated in 96-well plates

and exposed to the indicated strains of *C. neoformans* at an MOI of 1 for 24 hours. At that point, the supernatants were collected, centrifuged, and passed through a 0.2μm filter. The cell-free supernatant was then probed by an enzyme immunoassay (ELISA) to quantify the levels of secreted IL-1β and MIP-1β. The ELISA kits were obtained from bio-techne, an R&D Systems company.
